# Obese Children with Metabolic Syndrome Have 3 Times Higher Risk to Have Nonalcoholic Fatty Liver Disease Compared with Those without Metabolic Syndrome

**DOI:** 10.1155/2017/2671692

**Published:** 2017-10-08

**Authors:** Dimitrios Papandreou, Mirey Karavetian, Zacharoula Karabouta, Eleni Andreou

**Affiliations:** ^1^Department of Health Sciences, CNHS, Zayed University, Abu Dhabi, UAE; ^2^2nd Department of Pediatrics, School of Medicine, Aristotle University of Thessaloniki, Thessaloniki, Greece; ^3^Department of Life and Health Sciences, University of Nicosia, Nicosia, Cyprus

## Abstract

**Background:**

The aim of this study was to investigate the relationship between metabolic syndrome (MS) and nonalcoholic fatty liver disease (NAFLD) in obese children. One hundred and twenty-five subjects aged 11-12 years old participated in the study.

**Methods:**

Anthropometric and biochemical indices were measured, including lipid and liver profile, blood glucose, serum insulin, and liver ultrasound.

**Results:**

Forty-four children (58.6%) were found to have MS. Insulin resistance was present in 78 (62.4%) children. Patients with MS were more likely to have NAFLD (*P* < 0.001). Children with NAFLD had significantly higher body mass index, waist circumference, triglycerides, fasting insulin, and lower high-density lipoprotein compared to patients with normal livers (*P* < 0.001). Insulin resistance was significantly higher in children with NAFLD (*P* < 0.001). Obese children presenting with MS were 3.01 (2.87–3.57, *P* < 0.002) times more likely to develop NAFLD compared to those without metabolic syndrome after adjustment of cofounders.

**Conclusions:**

Obese children with MS have a higher risk of developing NAFLD. Weight management and early prevention should be the first line of treatment to prevent any possible health issues later on.

## 1. Introduction

The prevalence of childhood obesity is rising at an alarming rate worldwide [1]. Childhood obesity is a major health issue, and it is dramatically increasing in Greece [2, 3]. Data reveals that hypertension, type 2 diabetes, nonalcoholic fatty liver disease, and dyslipidemia are more prevalent in young children in Greece [4–6].

Metabolic syndrome (MS) involves a group of factors that together may lead to an increased risk of possible future cardiovascular disease (CVD) and has been found to associate with insulin resistance (IR) and type 2 diabetes mellitus. MS can be diagnosed in children with abdominal obesity aged 10 years or older, alongside two or more other clinical features (i.e., elevated triglycerides, low HDL cholesterol, high blood pressure, and increased plasma glucose) [7]. To date, there is no formal definition for diagnosis of MS in children and adolescents. In 2005, Alberti and colleagues developed a global definition for MS in adults [8]. Two years later, a new definition from the International Diabetes Federation [7] was developed that included all previous studies.

Since the risk factors for MS start from the early years of life [9], prevention of MS during childhood will decrease the risk of having metabolic disturbances in adulthood [10]. Nonalcoholic fatty liver disease (NAFLD) represents a wide spectrum of liver disease; it begins with fatty liver, which can progress to advanced nonalcoholic steatohepatitis and, lastly, to cirrhosis [11]. Data has shown that IR and hyperinsulinemia have a central role in the pathogenesis of both MS and NAFLD. It has been found that NAFLD is characterized by clinical and laboratory data that is similar to diabetes and obesity; for example, it has been characterized by impaired insulin sensitivity and abnormalities in lipid metabolism, even in the presence of normoglycaemia and normal or moderately increased body weight [12].

The aim of our study was to investigate cardiometabolic risk factors such as MS, IR, and lipid profile and how they relate to NAFLD among obese children.

## 2. Methods

A total of 145 obese children, aging 11-12 years old, were recruited from the pediatric obesity clinic of a general hospital in Thessaloniki, Greece, from February 2008 until September 2011. All children were screened for parameters of NAFLD, including anthropometric and biochemical indices as well as liver enzymes. Detailed medical history of all children was taken by a pediatrician of the clinic. Children with clinical evidence of diabetes, cardiovascular disease, liver disease, and alcohol and/or drug use were excluded from the study (*n* = 25). Thus, 125 children met the criteria and provided assent in addition to parental consent. In addition, the study was approved by the ethical committee of the Aristotle University of Thessaloniki (AU 1152).

### 2.1. Anthropometric Examination

Height, weight, Tanner stage, and waist circumference (WC) were recorded in all children. The body weight was measured to the nearest 0.1 kg using a SECA 700 model-scale. Height was measured to the nearest 0.1 cm using a SECA stadiometer. Body mass index was calculated as (weight (kg)/height (m)^2^). Obesity was defined using the criteria from International Obesity Task Force (IOTF), adjusted to age and sex [13]. All children included in the study were at Tanner stage 3. Waist circumference was measured at the level of the umbilicus to the nearest 0.1 cm using a regular tape. Children were considered obese when the waist circumference (WC) was equal and/or above the 90th percentile for age, height and gender, or adult cut of point if lower [14].

Using a regular mercury sphygmomanometer and the appropriate cuff according to the size of the child's upper right arm, a certified pediatrician measured blood pressure (BP) in all children. Three readings were obtained, each with a 3-minute interval, and the mean value of the last two was used. Normal blood pressure was defined as systolic blood pressure (SBP) and/or diastolic blood pressure (DBP) in the <90th percentile for gender, age, and height. Hypertension was defined as SBP and/or DBP > 95th percentile, as described by the National High Blood Pressure Education Program [15].

### 2.2. Ultrasound Examination

Ultrasonography (US) was used to diagnose NAFLD by the same radiologist using a convex probe with Elegra-Siemens equipment. Using a scale from 0 to 3, subjects were classified by severity of their hepatic steatosis, which ranged from normal, mild, moderate to severe. Classification was based on echogenicity and visualization of the vasculature, parenchyma, and diaphragm and compared to histological features [16]. The echogenicity of the liver was estimated in a longitudinal US slice, which showed the liver parenchyma and the neighboring right kidney. The echogenicity of the liver was compared to that of the adjacent kidney. Below is the detailed scaling scheme used in this study for NAFLD.

Scale 0: Normal: the echogenicity of the liver is minimally hyperechoic or isoechoic to normal renal cortex.
Scale 1: Mild: there is a slight increase of liver echogenicity, with normal visualization of the diaphragm and intrahepatic vessel borders.
Scale 2-3: Moderate to severe: The fatty liver has increased echogenicity and attenuation of the ultrasound beam. There is also impairment of visualization of the intrahepatic vessels, diaphragm, and the posterior right lobe of the liver [17].

### 2.3. Biochemical and Liver Profile

Fasting serum total cholesterol (TC), high-density lipoprotein (HDL), and triglyceride (TG) levels were measured using standard laboratory methods (Roche Diagnostics), while low-density lipoprotein (LDL) cholesterol was calculated using Friedwald's equation [18]. Normal serum levels of alanine aminotransferase (ALT) and aspartate aminotransferase (AST) were set at <40 U/l and <37 U/l, respectively. Fasting serum insulin measurement was done by an autoanalyzer (DDC/immulite). IR was quantified using HOMA (HOMA-IR) [19]:

(1) $$\begin{array}{c} Fasting\;serum\;insulin\;\left(uU/ml\right)\times \frac{fasting\;plasma\;glucose\;\left(mg/dl\right)}{405}. \end{array}$$

### 2.4. Metabolic Syndrome

Patients were labeled as having MS when three or more of the following modified criteria were present:

1. WC ≥ 90th percentile or adult cutoff if lower
2. Fasting blood glucose levels ≥ 100 mg/dl
3. TG levels ≥ 150 mg/dl
4. HDL ≤ 40 mg/dl
5. Blood pressure: systolic ≥ 130/diastolic ≥ 85 mm Hg [7]

### 2.5. Statistical Methods

SPSS version 21.0 was used. Data were summarized as mean and standard deviation (SD). Categorical data was examined by chi-square test, while differences in clinical and biochemical characteristics for continuous variables were tested by independent *t*-test. To assess the relationship between IR and the continuously distributed individual components of MS, Spearman's correlation coefficient was calculated. Logistic regression analysis was used using 3 different models to access the effect of MS on NAFLD in the obese subjects. *P* values < 0.05 were considered statistically significant.

## 3. Results

Anthropometric and clinical characteristics of all 125 subjects are presented in Table 1. ALT and AST were normal in all children. All children were obese based on criteria from IOTF. IR was present in 78 children (62.4%).

Additionally, 75 out of 125 (60%) patients had NAFLD with 44 children (58.6%) of them having three or more criteria of MS. All subjects had abdominal obesity (100%), 50.3% low HDL, 40% hypertriglyceridemia, 10.6% high fasting blood glucose, and 18.6% high blood pressure (Table 2).

Table 3 presents the characteristics of children with and without NAFLD. BMI, WC BP, TG, fasting insulin, and IR were found at significantly higher levels (*P* < 0.001) in children with NAFLD compared to children without. In addition, HDL levels were significantly (*P* < 0.001) lower in children with NAFLD, and in children presenting with MS, there was a significantly higher incidence of NAFLD (58.6% versus 10% *P* = 0.001).

In a logistic regression model, obese children presenting with MS were 3.01 (2.87–3.57, *P* < 0.002) times more likely to develop NAFLD compared to those without metabolic syndrome after adjusting for cofounders (Table 4).

## 4. Discussion

In this study, patients with NAFLD had clinically higher ALT levels compared to those with normal livers, but the difference was not statistically significant. It is known that many obese children still show normal ALT levels together with incipient evidence of a fatty liver [20, 21]. Obesity is strictly related to the development of NAFLD. In the present study, 75 out of 125 (60%) patients had NAFLD, which is similar to values observed by other authors [22–24].

In the present study, 75% of children with NAFLD also had a higher WC. Waist circumference has been associated with visceral fat, which is a precursor for the development of fatty liver disease [25] and CVD in children [26].

Insulin resistance has been found to increase lipolysis and free fatty acid output, which results in accumulation of TG within the hepatocytes [27]. We have shown in our study that fasting serum insulin was significantly higher in patients with NAFLD. This should be a cause for concern, since the high number of obese children with IR and NAFLD that meet MS criteria may have an increased risk of developing type 2 diabetes later on.

Many authors have reported the impact of MS as a risk factor for cardiovascular disease and the role of IR as a cause of MS. Metabolic syndrome has an increased risk of CVD independent of traditional risk factors in many large population-based studies [28–30]. Our data has shown that subjects with 3 or more cardiovascular risk factors (MS), as well as high insulin levels, have an increased risk for developing NAFLD even after adjusting for different confounders.

Limitations of the study are the nonusage of biopsy for NAFLD, which is the gold standard method. Also, the sample size was small. In addition, we did not take into consideration genetic, dietary, and activity factors that could have helped us further understand the relationship between MS, insulin resistance, and obesity in children.

## 5. Conclusions

Metabolic syndrome, insulin resistance, and fatty liver disease are inter-related to each other. Obese children with MS have a higher risk of developing NAFLD. More research is needed to elucidate the effects of MS in the development of NFLD in obese children and the possible health problems that will face on later in their lives.

## Figures and Tables

**Table 1:** Anthropometric and biochemical characteristics of the 125 obese patients.

Characteristics	Subjects (*n* = 125)	Number of patients with abnormal results (%)
*Anthropometric*		
Sex (male/female)	60/65	
Age (years) (mean ± SD)	12.3 ± 1.1	
Overweight/obese	0/125	
Waist circumference (cm)	97 ± 5.88	125 (100)
Body mass index (kg/m^2^)	33 ± 7.89	125 (100)
Systolic blood pressure (mm/Hg)	103 ± 9.5	25 (20)
Diastolic blood pressure (mm/Hg)	77 ± 7.8	17 (13.6)
*Biochemical*		
ALT (U/l)	24 ± 8	0 (0)
AST (U/l)	22 ± 9	0 (0)
Total cholesterol	162 ± 28.1	0 (0)
LDL (mg/dl)	98 ± 2.5	0 (0)
HDL (mg/dl)	44 ± 9.3	45 (36)
Non-HDL (mg/dl)	139 ± 29.2	52 (41.6)
Triglyceride (mg/dl)	142 ± 45	50 (40)
Fasting blood glucose (mg/dl)	94.5 ± 6.8	12 (9.6)
Fasting insulin level (uU/ml)	33.7 ± 7.9	
HOMA-IR	7.8 ± 5.7	78 (62.4)

Data is presented as mean ± SD. LDL: low-density lipoprotein; HDL: high-density lipoprotein.

**Table 2:** Frequency at which criteria for MS were met in patients with NAFLD (*n* = 75).

Variable	(*n*/%)
Waist circumference ≥ 90th percentile	75 (100)
HDL ≤ 40 mg/dl	38 (50.6)
Triglycerides ≥ 110 mg/dl	30 (40)
Fasting blood glucose ≥ 100 mg/dl	8 (10.6)
Blood pressure ≥ 130/85 mm Hg	14 (18.6)

MS: metabolic syndrome; HDL: high-density lipoprotein.

**Table 3:** Characteristics of children with and without nonalcoholic fatty liver disease (NAFLD).

Variable	NL (*n* = 50)	NAFLD (*n* = 75)	*P* value
Sex (M/F)	25/25	35/40	0.393
Age (years)	12.1 ± 1.1	12.2 ± 1.2	0.534
Obesity	25/25	35/40	0.454
BMI (kg/m^2^)	30 ± 3.9	33 ± 5.2	0.001^∗^
WC (cm)	87 ± 12	97 ± 11	0.001^∗^
SBP (mm/Hg) DBP (mm/Hg)	94 ± 7.3	97 ± 7.1	0.689
ALT (U/l)	22.7 ± 6.2	26 ± 8.1	0.512
AST (U/l)	21.1 ± 7.4	25 ± 8.2	0.443
Total cholesterol (mg/dl)	165 ± 27	168 ± 29	0.654
LDL (mg/dl)	104 ± 20	105 ± 21	0.887
HDL (mg/dl)	51 ± 10	42 ± 873	0.001^∗^
Non-HDL (mg/dl)	114 ± 25	141 ± 21	0.001^∗^
Triglycerides (mg/dl)	134 ± 53	170 ± 30	0.001^∗^
Fasting blood glucose (mg/dl)	99.4 ± 7.2	88.8 ± 8.4	0.441
Fasting blood insulin (uU/ml)	25.2 ± 6.2	38.7 ± 8.4	0.001^∗^
HOMA-IR	6.18 ± 1.1	8.49 ± 1.1	0.001^∗^
MS (≥3 criteria)	5 (10%)	44 (58.6)	0.001^∗^

^∗^ *P* < 0.05 (statistically significant). WC: waist circumference; LDL: low-density lipoprotein; HDL: high-density lipoprotein; HOMA-IR: insulin resistance as assessed by homeostasis model. Data is presented as mean ± SD.

**Table 4:** Logistic regression model for NAFLD in obese children with and without metabolic syndrome.

MS (*n* = 44) versus NMS (*n* = 81)		
	OR (95% CI)	*P*
Model 1	5.41 (3.94, 5.89)	0.001^∗^
Model 2	4.34 (3.88, 4.88)	0.001^∗^
Model 3	3.01 (2.87, 3.57)	0.002^∗^

^∗^ *P* < 0.05 (statistically significant). OR: odds ratio, CI: confidence interval. Model 1: unadjusted effects; Model 2: adjusted for age and gender; Model 3: adjusted for age, gender, LDL, and insulin resistance.
